# Field-Based Performance Tests Are Related to Body Fat Percentage and Fat-Free Mass, But Not Body Mass Index, in Youth Soccer Players

**DOI:** 10.3390/sports6040105

**Published:** 2018-09-27

**Authors:** Michael R. Esco, Michael V. Fedewa, Zackary S. Cicone, Oleg A. Sinelnikov, Damir Sekulic, Clifton J. Holmes

**Affiliations:** 1 Department of Kinesiology, University of Alabama, Box 870312, Tuscaloosa, AL 35487, USA; mvfedewa@ua.edu (M.V.F.); zcicone@crimson.ua.edu (Z.S.C.); osinelnikov@ua.edu (O.A.S.); cjholmes2@crimson.ua.edu (C.J.H.); 2 Faculty of Kinesiology, University of Split, 21000 Split, Croatia; damirsekulich@gmail.com

**Keywords:** performance, soccer, youth athletes, body composition

## Abstract

The primary aim of this study was to determine the association between body composition and performance outcomes in youth soccer players. Twenty-five competitive male youth soccer players (age = 13.7 ± 0.8 years, height = 167.4 ± 9.7 cm, weight = 57.6 ± 12.1 kg) volunteered to participate in this study. Height and weight were used to calculate body mass index (BMI). Body fat percentage (BF%) and fat-free mass (FFM) were determined with dual-energy X-ray absorptiometry. Each athlete performed the Pacer test, vertical jump, and *t*-test drill. Participants were predominantly normal weight (20.4 ± 2.7 kg·m^−2^). The body composition results were 20.3 ± 4.9% for BF% and 46.5 ± 8.7 kg for FFM. The results of the performance tests indicated a mean ± standard deviation (SD) of 1418 ± 332 m for Pacer, 57.2 ± 7.4 cm for vertical jump, 11.6 ± 0.7 s for *t*-test. Body mass index was not associated with any performance measure (r = 0.02 to −0.21, all *p* > 0.05). Body fat percentage was associated with the Pacer, vertical jump, and *t*-test (r = −0.62, −0.57, 0.61, respectively; all *p* < 0.01) and remained after accounting for BMI. Fat-free mass was only significantly related to *t*-test (r = −0.43, *p* < 0.01). However, after controlling for BMI, FFM was related to all three performance tests. Body fat percentage and FFM are associated with performance in youth soccer players, with stronger relationships reported in the former metric. The findings highlight the need for accurate body composition measurements as part of an assessment battery in young athletes.

## 1. Introduction

Determining the physiological factors that influence soccer performance has been a focus of a wide body of research. Studies have indicated that soccer athletes tend to display competencies across a range of physical fitness parameters [1,2]. However, aerobic fitness, agility, and explosive power in the lower body appear to be some of the most important physical capacities regarding on-field soccer performance [3]. Indeed, each of these fitness metrics have been shown to be related to skill-level, with studies indicating superior aerobic capacity, agility, and explosive lower body power in better performing players [3,4,5]. Therefore, determining the trainable characteristics that correlate to these three performance qualities is an important consideration in sport science.

Body composition also appears to be an important fitness parameter concerning soccer-related performance. Previous work has indicated that soccer players tend be significantly leaner compared to non-athletes [6]. Additionally, body fat percentage (BF%) has been shown to correlate with aerobic and anaerobic fitness capacities [7,8,9,10], while fat-free-mass (FFM) strongly relates to movements that involve rapid skeletal muscle activation [11]. Importantly, both metrics appear to be significant contributors to the three fitness parameters mentioned in the previous paragraph (i.e., aerobic fitness, agility, and explosive lower body power) in adult soccer players [9]. Findings such as these underscore the importance of decreasing BF% and increasing FFM throughout a period of conditioning.

Body mass index (BMI) is perhaps the most popular method of body mass stature in children. However, whether it is related to performance in young soccer players is less clear. With respect to the limited research, Nikolaidis [12] showed that both BMI and BF% were related to tests of aerobic and anaerobic capacities in youth soccer players aged 12 to 14 years. However, stronger relationships to the performance markers were displayed with the latter body composition measure. Though the findings of the study were novel, a few concerns were present that warrant further investigation. For instance, not all of the laboratory tests of aerobic fitness and anaerobic power were ecologically valid, BF% was evaluated with a skinfold prediction technique, and FFM was not studied [12]. Further research is needed to expand on previous findings by using a more sophisticated and robust measure of body composition and field-based tests for analyzing performance that are familiar to younger athletes. The evident lack of research on the association between body composition and performance in youth soccer is additionally important knowing that body composition parameters are relatively trainable. In other words, BMI, BF% and FFM may be efficiently modified throughout appropriate training methods [13,14]. Information on associations that may exist between body composition and different performance indicators in youth soccer players could be important from the viewpoint of long-term athletic development.

The primary aim of this study was to determine the associations between body composition and performance variables in youth soccer players. It was hypothesized that higher BMI and BF% would be associated with poorer performance on each of the performance metrics, whereas higher FFM would be associated with more favorable performance on each of the performance tests. A secondary aim of this study was to determine if BF% and FFM were better predictors of performance when compared to BMI. The hypothesis was that FFM and BF% would explain a greater proportion of performance than BMI alone.

## 2. Methods

### 2.1. Participants

Twenty-five competitive male youth soccer players (age = 13.7 ± 0.8 years, height = 167.4 ± 9.7 cm, weight = 57.6 ± 12.1 kg) volunteered to participate in this study. All participants were informed of the requirements of the investigation. Each participant and their parent or guardian provided written consent to participate. Testing occurred at the beginning of the competitive season. The participants were asked to attend each testing session in a normally fed and hydrated state. The investigation was approved by the University of Alabama Institutional Review Board.

### 2.2. Body Composition

Body composition measurements were performed in the Exercise Physiology Laboratory at the University of Alabama. On arrival at the laboratory, weight was measured (to the nearest 0.1 kg) with a calibrated digital scale (Tanita BWB-800, Tanita Corporation, Tokyo, Japan) followed by height (to the nearest 0.1 cm) with a stadiometer (SECA 213, Seca Ltd., Hamburg, Germany). Total body dual energy X-ray absorptiometry (DXA) scans were then performed with a GE Lunar Prodigy (Software version 10.50.086, GE Lunar Corporation, Madison, WI, USA) to measure BF% and FFM. Before each scan, the DXA was calibrated according to the manufacturer’s instructions using the standard calibration block. While wearing a T-shirt and shorts without any metal, each participant was instructed to lie motionless in a supine position on the scanning bed with their arms by the sides and palms in a neutral position with knees and ankles held together with a Velcro strap.

### 2.3. Performance Tests

The Pacer test was performed on an outdoor soccer practice field. The test required participants to shuttle-run back and forth between 2 markers placed 20 m apart. The required speed of each subsequent stage became progressively faster. The test commenced at a speed of 8.0 km·h^−1^, increased to 9.0 km·h^−1^ for the second stage, and thereafter increased by 0.5 km·h^−1^ each minute per stage. Pace was maintained by audible cues (loud beeps) that were delivered from a smartphone application. The test was terminated when the participant could no longer maintain the required pace, which was evidenced by failure to reach the appropriate 20-m mark on 2 consecutive occasions. The maximal achieved distance in meters was recorded as the indicator of shuttle-run performance. The participants performed the Pacer in groups of 4–8 to stimulate a competitive environment and were familiar with the test as it was performed as a regular part of their training.

The participants were measured for their vertical jumping ability using the Vertec apparatus (Questek, Sports Imports, Hilliard, OH, USA) on an indoor wooden court. Before performing the vertical jump test, the participants performed 5 squat jumps at a submaximal intensity. The Vertec involved a sliding vertical pole with attached movable perpendicular vanes separated by 1.27 cm. Maximal reach was determined, with the participants placing both feet flat on the ground and reaching as high as possible with the dominant hand placed on the vertical pole. The height of the pole was adjusted so that the participant’s maximal reach was touching a red marking placed 30.48 cm below the lowest vane. Once the pole was situated, the participants were instructed to perform the vertical jump. A countermovement was allowed in which the participant began in an upright posture with their feet shoulder width apart. The participant then moved into a partial squat position while swinging their arms back to prepare for the jump. Then, the arms quickly moved forward above their head as they extended their hips and knees while jumping vertically into the air. The participants reached as high as possible with their dominant hand to move as many levers as possible at the top portion of the jump. The height of the jump was determined as the difference between the predetermined vertical reach and the height of the vertical jump. Three vertical jumps were performed and the highest jump was recorded.

The *t*-test agility drill was performed on an indoor court with timing by two technicians holding stopwatches. Before testing, the participants performed a dynamic warm-up drill that consisted of several shuttle runs over a 20 m distance interspersed with 30 s at the completion of each shuttle. The warm-up drill lasted approximately 5 min. The *t*-test drill began with the participants standing with both feet behind the starting point. At their own discretion, each participant sprinted forward 9.14 m and touched the base of the center cone with their dominate hand. Then, they shuffled to the left 4.52 m and touched the base of the left outside cone with their left hand. Participants then shuffled to the right 9.14 m and touched the base of the outside right cone with their right hand. Following that, they shuffled 4.57 m to the left, back to the center cone and touched its base. Participants then ran backward, passing the finishing line at the first cone. Two trials were performed at a submaximal level of exertion for familiarization. For testing purposes, three test trials were performed with times recorded to the nearest one-hundredth with a stopwatch by one of two trained technicians. Both technicians had experience of implementing the *t*-test drill, one being a Certified Strength and Conditioning Specialist and the other a certified youth soccer coach. Though a concern for error may exist with manual timing, previous research has indicated that timing measurements by a trained rater using a stopwatch are valid for assessing agility in adolescents when compared to digital timing systems [15].

### 2.4. Statistics

Statistical analysis was performed using a Statistical Package for the Social Sciences or SPSS (SPSS 23.0, IBM Corp, Armonk, NY, USA). Data were screened for outliers, with skewness or kurtosis >2 indicating non-normal distribution. There were no outliers present in the study. Bivariate correlations were assessed using Pearson’s r to determine the strength and direction of the association between the anthropometric (BMI), body composition (BF% and FFM) and performance variables (Pacer, vertical jump, and *t*-test). The alpha level of significance was predetermined as *p* < 0.05. Hopkins scale was used to qualify the correlation coefficients as follows: r = 0.0–0.1 as trivial; 0.1–0.3 as small; 0.3–0.5 as moderate; 0.5–0.7 as large; 0.7–0.9 as very large; >0.9 as near perfect [16]. A secondary aim of this study was to determine the additional variation in performance that can be explained by body composition, beyond BMI alone. Multivariate linear regression quantified the associations of the independent variables of BMI, %BF, and FFM with the performance variables (Pacer, vertical jump, *t*-test). Model fit was assessed using the R^2^ and R^2^ change (ΔR^2^) statistics, beginning by comparing a univariate model containing only BMI with two separate models including %BF and FFM for each performance test. All data are expressed as mean ± standard deviation (SD), unless otherwise indicated.

## 3. Results

The mean ± SD results for the body composition and performance results are shown in Table 1. Pearson correlations demonstrated the following non-significant correlations between BMI and the performance tests: r = −0.20 (*p* = 0.33, small) for Pacer; r = −0.12 (*p* = 0.58, small) for *t*-test; and r = 0.02 for vertical jump (*p* = 0.93, trivial). The significant correlations between BF% and the *t*-test was r = 0.61 (*p* < 0.01, large), Pacer was r = −0.62 (*p* < 0.01, large), and vertical jump was r = −0.57 (*p* < 0.01, large). The only significant correlate to FFM was *t*-test (r = −0.43, *p* = 0.03, moderate). Fat-free mass did not significantly correlate with vertical jump (r = 0.30, *p* = 0.24, moderate) or Pacer (r = 0.18, *p* = 0.40, small).

The results of the linear regression procedures are shown in Table 2 and Table 3. The regression model containing only BMI explained a small amount of the variation in Pacer, vertical jump, and *t*-test performance (R^2^ = 4.1%, 0.0%, 1.3%, respectively; all *p* > 0.05). Including BF% as an additional independent variable significantly increased the model R^2^ for Pacer (ΔR^2^ = 35.8%, *p* = 0.002), vertical jump (ΔR^2^ = 47.3%, *p* < 0.001), and *t*-test (ΔR^2^ = 62.2%, *p* < 0.001) (Table 2). Similarly, including FFM as an additional independent variable improved model fit for Pacer (ΔR^2^ = 21.8%, *p* = 0.019), vertical jump (ΔR^2^ = 17.7%, *p* = 0.041), and *t*-test (ΔR^2^ = 24.7%, *p* = 0.013) (Table 3).

## 4. Discussion

This investigation sought to determine relationships between body composition and performance variables in youth soccer players. The performance indicators were chosen due to their being ecologically valid tests and hence commonly employed for assessing aerobic fitness (Pacer), agility (*t*-test), and explosive lower body power (vertical jump) in sportive settings. The body composition parameters were BMI, BF% and FFM, with the latter two measures being analyzed with DXA. Previous studies examining relationships between performance and body composition in youth athletes used prediction methods of BF%, such as the skinfold technique [12,17]. Compared to the skinfold method, DXA is a laboratory technique that has been considered by some authors to be the “gold standard” for body composition assessment in young subjects [18]. To our knowledge, this study is the first to utilize DXA for measuring BF% and FFM for determining their associations with performance markers in youth soccer players when compared to BMI alone.

The findings of the correlation procedures suggested that BMI was not significantly related to any performance test. Body fat percentage was significantly and negatively correlated with Pacer and vertical jump scores and positively correlated to the *t*-test drill. The only significant correlation to FFM was a negative association with *t*-test. Fat-free mass was not significantly correlated with Pacer or vertical jump. However, when controlling for BMI with the regression procedures, the significant relationships with the performance tests remained for BF% and were revealed (for all three performance tests) for FFM. The regression analyses indicated that a greater amount of variation in performance can be explained by including a body composition estimate, beyond what can be explained by BMI alone. These results indicate that after accounting for the variation explained by BMI, BF% and FFM were associated with each performance test. Therefore, the hypothesis that each body composition metric would be significantly correlated to the performance was partially accepted.

Body mass index has become the primary method to establish the standards for and determine prevalence of overweight and obesity in public health and clinical settings [19,20]. However, because BMI does not distinguish between fat and fat-free tissues, it is not a useful body composition measure in athletes who tend to display lower BF% and greater FFM at any given BMI compared to non-athletes [21,22]. Thus, BMI often misclassifies lean athletes with greater FFM as overweight [21,22]. Consequently, BMI has been shown to be a poor indicator of BF% or FFM in youth athletes [23,24]. However, BMI was associated with running and jumping performance in a sample of 72 youth basketball players (r = 0.24–0.42) [25]. The discrepant findings compared to the current study are likely related to a different group of studied athletes (basketball versus soccer) and different tests of performance.

The current results are consistent with other investigations that indicated body composition measures may serve as better predictors of performance than BMI in young athletes. For instance, two previous studies showed that BF% was a better correlate to tests of aerobic and anaerobic fitness capacities than BMI in youth soccer [12] and volleyball [17] players. In the previous study of young basketball players, BF% was also correlated with running and jumping performance; however, it was not directly compared to BMI [25]. Collectively, the current and previous results suggest that elevated BF% may negatively impact performance in youth athletes, and may serve as a better predictor of performance when compared to BMI.

Increased BF% presents the addition of non-metabolically active tissue that provides an inertial challenge and does not contribute to physiological capacity to produce force. Thus, excess adiposity may impede muscular performance and slow movement in young athletes. Independent of body size, higher BF% may decrease the ability to sustain the repeated intermittent bouts of high-intensity exertion across the duration of a soccer game [1] and may result in less playing opportunities for individual athletes. On the other hand, lower BF% may be associated with delayed onset of fatigue and allow the ability to sustain the combined aerobic and anaerobic activities of soccer-play for longer duration. Therefore, the focus of conditioning programs should be placed on achieving or maintaining an optimal level of BF% at any given body mass.

Fat-free mass is also an important body composition metric in regards to sport performance. In adult soccer players, higher performing athletes have been shown to display greater FFM values compared to lower level performers [26]. However, the associations between FFM and the three performance measures used in the current study was not previously determined in youth soccer athletes. Fat-free mass was associated with all performance indicators in the current study after controlling for BMI. However, it should be noted that the associations between FFM and performance indicators were slightly weaker than the associations between BF% and performance in the current sample. Indeed, these results demonstrate the need for additional research to determine the specific physiological variables and trainable characteristics needed to improve performance in youth soccer players and should include other variables related to muscular performance that were not analyzed in the current study.

There are some limitations with this study that should be highlighted. First, the cross-sectional nature of the study does not allow one to infer a cause-and-effect. Thus, it is unclear whether decreases or increases in BMI, BF% or FFM directly influence performance results. Second, only male subjects aged 13 to 15 were studied, which prevents generalizing the results to females and youth subjects from older or younger age groups. Third, compared to FFM, neuromuscular and strength indicators may have provided better insight into the role that skeletal muscle fitness contributed to the performance tests. Though the focus of the study was the relationship between body composition and performance, future research should include neuromuscular markers when determining predictors of soccer-related performance in youth athletes. Fourth, though the sample was of sufficient size to pursue the correlation analyses, it was too small to divide the group according to playing position. This is an important consideration as differences in performance and body composition across playing positions have been reported [27]. Larger sample sizes in future research will be needed to better understand position-specific relationships. Last, the duration of training background for the participants was not recorded, which may have influenced the results and hence warrants further research.

## 5. Conclusions

This study found that BF% and FFM were associated with three field-based performance tests (Pacer, *t*-test, and vertical jump) after accounting for the variation explained by BMI. In fact, BMI failed to display any significant bivariate correlations with the three performance tests. Because soccer requires the athletes to sustain highly intense levels of intermittent exertion across a lengthy period of time during play, higher BF% and lower FFM may be associated with decreased performance and result in a faster onset of fatigue. Strength and conditioning professionals are encouraged to include a measure of body composition as part of their assessment battery in young athletes, as well as develop programs that not only focus on sport-specific activities but are also designed to achieve optimal body composition at any given body mass. Practitioners are encouraged to follow standard practices when prescribing exercise to youth athletes and avoid focusing on hypertrophy or achieving increased body mass during developmental years. However, future studies are needed to confirm these results.

## Figures and Tables

**Table 1:** Mean ± standard deviation (SD) of the studied variables.

Variable	Mean ± SD
BMI (kg·m^−2^)	20.4 ± 2.7
BF% (%)	20.3 ± 4.9
FFM (kg)	46.5 ± 8.7
Pacer (m)	1418 ± 332
Vertical jump (cm)	57.2 ± 7.4
*t*-test (s)	11.6 ± 0.7

BMI = body mass index, BF% = body fat percentage, FFM = fat-free-mass.

**Table 2:** Hierarchical linear models of body mass index, relative adiposity, and performance measures in youth soccer athletes.

Variable		Model 1—Only BMI as the Variable			Model 2—BMI and BF% as the Variables		
		Parameter Estimate	SE	*p* Value	Parameter Estimate	SE	*p* Value
Pacer							
	Intercept	1924.04	517.09	0.001	1949.54	418.65	<0.001
	BMI	−24.82	25.17	0.334	22.14	24.16	0.369
	%BF	-	-	-	−46.36	12.81	0.002
	R^2^	0.04	-	0.334	0.40	-	0.004
	ΔR^2^	-	-	-	0.36	-	0.002
Vertical jump							
	Intercept	56.22	11.85	0.001	56.88	8.79	<0.001
	BMI	0.05	0.58	0.931	1.26	0.51	0.021
	%BF	-	-	-	−1.20	0.27	<0.001
	R^2^	0.00	-	0.931	0.47	-	0.001
	ΔR^2^	-	-	-	0.47	-	<0.001
*t*-test							
	Intercept	12.23	1.15	<0.001	12.16	0.71	<0.001
	BMI	−0.03	0.06	0.581	−0.17	0.04	0.021
	%BF	-	-	-	0.13	0.02	<0.000
	R^2^	0.01		0.581	0.64	-	<0.001
	ΔR^2^	-	-	-	0.62	-	<0.001

Note: BMI = body mass index; %BF = relative adiposity; SE = standard error.

**Table 3:** Hierarchical linear models of body mass index, fat-free mass, and performance measures in youth soccer athletes.

Variable		Model 1—Only BMI as the Variable			Model 2—BMI and FFM as the Variables		
		Parameter Estimate	SE	*p* Value	Parameter Estimate	SE	*p* Value
Pacer							
	Intercept	1924.04	517.09	0.001	1949.12	464.92	<0.001
	BMI	−24.82	25.17	0.334	−85.04	32.76	0.016
	FFM	-	-	-	25.87	10.18	0.019
	R^2^	0.04	-	0.334	0.26	-	0.019
	ΔR^2^	-	-	-	0.22	-	0.037
Vertical jump							
	Intercept	56.22	11.85	0.001	56.73	10.99	<0.001
	BMI	0.05	0.58	0.931	−1.17	0.77	0.146
	FFM	-	-	-	0.52	0.24	0.041
	R^2^	0.00	-	0.931	0.18	-	0.118
	ΔR^2^	-	-	-	0.18	-	0.041
*t*-test							
	Intercept	12.23	1.15	<0.001	12.18	1.01	<0.001
	BMI	−0.03	0.06	0.581	0.11	0.07	0.142
	FFM	-	-	-	−0.06	0.02	0.013
	R^2^	0.01	-	0.581	0.26	-	0.036
	ΔR^2^	-	-	-	0.25	-	0.013

Note: BMI = body mass index; FFM = fat-free mass; SE = standard error.

## References

[1] Bangsbo J., Mohr M., Krustrup P. (2006). Physical and metabolic demands of training and match-play in the elite football player. J. Sport Sci..

[2] Vicente-Rodríguez G., Rey-López J.P., Ruíz J.R., Jiménez-Pavón D., Bergman P., Ciarapica D., Heredia J.M., Molnar D., Gutierrez A., Moreno L.A. (2011). Interrater reliability and time measurement validity of speed–agility field tests in adolescents. J. Strength Cond. Res..

[3] Fiorilli G., Mitrotasios M., Iuliano E., Pistone E.M., Aquino G., Calcagno G., DI Cagno A. (2016). Agility and change of direction in soccer: Differences according to the player ages. J. Sport Med. Phys. Fit..

[4] De Gouvêa M.A., Cyrino E.S., Valente-dos-Santos J., Ribeiro A.S., da Silva D.R.P., Ohara D., Coelho-e-Silva M.J., Ronque E.R.V. (2017). Comparison of skillful vs. Less skilled young soccer players on anthropometric, maturation, physical fitness and time of practice. Int. J. Sports Med..

[5] Witt K.A., Bush E.A. (2005). College athletes with an elevated body mass index often have a high upper arm muscle area, but not elevated triceps and subscapular skinfolds. J. Am. Diet. Assoc..

[6] Portal S., Rabinowitz J., Adler-Portal D., Burstein R.P., Lahav Y., Meckel Y., Nemet D., Eliakim A. (2010). Body fat measurements in elite adolescent volleyball players: Correlation between skinfold thickness, bioelectrical impedance analysis, air-displacement plethysmography, and body mass index percentiles. J. Pediatr. Endocr. Met..

[7] Chaouachi A., Brughelli M., Chamari K., Levin G.T., Abdelkrim N.B., Laurencelle L., Castagna C. (2009). Lower limb maximal dynamic strength and agility determinants in elite basketball players. J. Strength Cond. Res..

[8] Miller D.K., Kieffer H.S., Kemp H.E., Torres S.E. (2011). Off-season physiological profiles of elite national collegiate athletic association division iii male soccer players. J. Strength Cond. Res..

[9] Tangalos C., Robertson S.J., Spittle M., Gastin P.B. (2015). Predictors of individual player match performance in junior australian football. Int. J. Sport Physiol..

[10] Alemdaroğlu U. (2012). The relationship between muscle strength, anaerobic performance, agility, sprint ability and vertical jump performance in professional basketball players. J. Hum. Kinet..

[11] Deprez D., Valente-dos-Santos J., Silva M., Lenoir M., Philippaerts R., Vaeyens R. (2015). Multilevel development models of explosive leg power in high-level soccer players. Med. Sci. Sport Exerc..

[12] Nikolaidis P.T. (2012). Elevated body mass index and body fat percentage are associated with decreased physical fitness in soccer players aged 12–14 years. Asian J. Sports Med..

[13] Cuddy J.S., Slivka D.R., Hailes W.S., Ruby B.C. (2011). Factors of trainability and predictability associated with military physical fitness test success. J. Strength Cond. Res..

[14] Marta C.C., Marinho D.A., Barbosa T.M., Carneiro A.L., Izquierdo M., Marques M.C. (2013). Effects of body fat and dominant somatotype on explosive strength and aerobic capacity trainability in prepubescent children. J. Strength Cond. Res..

[15] Wittich A., Oliveri M.B., Rotemberg E., Mautalen C. (2001). Body composition of professional football (soccer) players determined by dual X-ray absorptiometry. J. Clin. Densitom..

[16] Hopkins W., Marshall S., Batterham A., Hanin J. (2009). Progressive statistics for studies in sports medicine and exercise science. Med. Sci. Sport Exerc..

[17] Nikolaidis P.T. (2013). Body mass index and body fat percentage are associated with decreased physical fitness in adolescent and adult female volleyball players. J. Res. Med. Sci..

[18] Hussain Z., Jafar T., uz Zaman M., Parveen R., Saeed F. (2014). Correlations of skin fold thickness and validation of prediction equations using dexa as the gold standard for estimation of body fat composition in pakistani children. BMJ Open.

[19] NIH (1998). Clinical Guidelines on the Identification, Evaluation and Treatment of Overweight and Obesity in Adults: The Evidence Report.

[20] Saint-Maurice P.F., Welk G.J., Laurson K.R., Brown D.D. (2014). Measurement agreement between estimates of aerobic fitness in youth: The impact of body mass index. Res. Q. Exerc. Sport.

[21] Ode J.J., Pivarnik J.M., Reeves M.J., Knous J.L. (2007). Body mass index as a predictor of percent fat in college athletes and nonathletes. Med. Sci. Sport Exerc..

[22] World Health Organization (WHO) (1998). Obesity: Preventing and Managing the Global Epidemic: Report of a Who Consultation on Obesity.

[23] Silvestre R., West C., Maresh C.M., Kraemer W.J. (2006). Body composition and physical performance in men’s soccer: A study of a national collegiate athletic association division i team. J. Strength Cond. Res..

[24] Stølen T., Chamari K., Castagna C., Wisløff U. (2005). Physiology of soccer. Sports Med..

[25] Nikolaidis P.T., Asadi A., Santos E.J., Calleja-González J., Padulo J., Chtourou H., Zemkova E. (2015). Relationship of body mass status with running and jumping performances in young basketball players. Muscles Ligaments Tendons J..

[26] Micheli M.L., Pagani L., Marella M., Gulisano M., Piccoli A., Angelini F., Burtscher M., Gatterer H. (2014). Bioimpedance and impedance vector patterns as predictors of league level in male soccer players. Int. J. Sport Physiol..

[27] Cárdenas-Fernández V., Chinchilla-Minguet J.L., Castillo-Rodríguez A. (2017). Somatotype and body composition in young soccer players according to the playing position and sport success. J. Strength Cond. Res..

